# Supplementary material for: Patterns of Intron Gain and Loss in Fungi
Source: PLoS Biol. 2004 Nov 30;2(12):e422. doi: 10.1371/journal.pbio.0020422 (PMC532390; doi:10.1371/journal.pbio.0020422)
Supplement: Table S1 — Also available at http://genes.mit.edu/NielsenEtAl/. (4.3 MB ZIP). [file pbio.0020422.st001.zip › NielsenEtAl/html/760.html]

AN5217.1.NCU07495.1.MG00153.1.FG00900.1


```
 CLUSTAL W (1.82) Multiple Sequence Alignments - Introns Inserted


Sequence 1: MG00153.1	361 aa
Sequence 2: FG00900.1	352 aa
Sequence 3: NCU07495.1	396 aa
Sequence 4: AN5217.1	347 aa
Alignment Length: 397 aa
Number Identitical Residues: 229 aa
Alignment Score (without introns) 9693


MG00153.1 	MHRTFSMRHSRAPTASQLQ0NPPPPPSSTKSGRIFGRGAFG1---HALRRNAAGAFGPDL
NCU07495.1	MHRTYSMRASRAPTASQIQ~NPPPPPSSTKSGRLFGRGGIG1GLGHALRRNAAGAFGPDL
FG00900.1 	MHRTYSMRQTRAPTASQLQ0NPPPPPSSTKSGRLF-KGSFG1---HALRRNAGGAFGPDL
AN5217.1  	MHRTYSMRQSRLPTASQIE~NPPPPLSSTKTNRWMGKGGLG1---HAFRKNAAGAFGPDL
          	****:*** :* *****:: ***** ****:.* :.:*.:*    **:*:**.*******

MG00153.1 	AKKLSQLVKMEKNVMRSLEQVARERMEVA0QQLSLWGEHGDEDVSDVTDKLGVLLYEIGE
NCU07495.1	AKKLSQLVKMEKNVMRSLEMVAKERMEVA~QQLSLWGEACDEDVSDVTDKLGVLLYEIGE
FG00900.1 	AKKLSTLVKMEKNVMRSLETVARERMEVA0QQLSIWGEAGDEDVSDVTDKLGVLVYEIGE
AN5217.1  	ARKLSQLVKMEKNVMRSMELVSRERMETA0QQLSIWGENCDEDVSDITDKLGVLLYEVGE
          	*:*** ***********:* *::****.* ****:***  ******:*******:**:**

MG00153.1 	LEDQYVDRYDQYRLTMKSIR~NIEASVQPSRD----------------------R1KQKI
NCU07495.1	LEDQYVDRYDQYRVTMKSIR~NIEASVQPSRDSRSTLLLLLSQLLSLFTTNTPRG~KQKI
FG00900.1 	LEDQYVDRYDQYRVTMKSIH1SAHMHLLTSTT----------------------G~KQKI
AN5217.1  	LEDMYVDRYDQYRVTIKSIR~NIEASVQPSRD----------------------R1KQKI
          	*** *********:*:***: . .  : .*                          ****

MG00153.1 	TDQIAHLKYKEPNSPKIVVLEQELVRAEAESLVAEAQLSNITREKIKAAYAYQFDALREH
NCU07495.1	TDQIAQLKYKEPNSPRIVVLEQELVRAEAESLVAEAQLSNITREKIKAAYTYQFDALREH
FG00900.1 	TDQIAQLKYKEPQSPKIVVLEQELVRAEAESLVAEAQLSNITREKVKAAYTYQFDALREH
AN5217.1  	TDEIAKLKYKDPNSPRIVVLEQELVRAEAESLVAEAQLSNITREKLKAAFQYQFDALREH
          	**:**:****:*:**:*****************************:***: *********

MG00153.1 	SEKVAIIAGYGKHLLELIDDTPVTPGETRAAYDGYEASKAIIQDCEDALTGWVTQNAAVS
NCU07495.1	CEKVAIIAGYGKHLLELIDDTPVTPGETRPAYDGYEASKAIIQDCEDSLTNWVTQNAAVS
FG00900.1 	CEKVAIIAGYGKHLLELIDDTPVTPGETRQAYDGYEASKAIIQDCEDALTSWVSSNAVVS
AN5217.1  	CEKVAIIAGYGKHLLDLIDDTPVTPGETRQAYDGYDASKAIIQDCEDALANWVSSKAAVK
          	.**************:************* *****:***********:*:.**:.:*.*.

MG00153.1 	SKLSTRARTLSTRRRNHIKARAEGG----HDLSAQDAPLN-EQGSWVQGSNVRGQDEFDD
NCU07495.1	AKLSTRSRTLSQRRRNNIKARTEGGAGQGHDLSGQDAPLN-DRDSWVPANQHKEVADYEV
FG00900.1 	SKLSTRARTLSQRRRNNIRTRGEG-----HDLSGQDMPLN-DGSSWT----ARDRD-LDS
AN5217.1  	SSLSQRSRTLSQRRQDNINKGRENG----VDLSSQDQPLRGDRDSWVA---AEQHPSYHH
          	:.** *:**** **:::*.   *..     ***.** **..: .**.     .   . . 

MG00153.1 	DEDELEDERDSS----ILDSENGLNGERE--RRGRSQEAVLA
NCU07495.1	SEEEEDDDEDEEDDIHASGSHSMLDGESEQHQRGRNTEPVAA
FG00900.1 	EEEEEDEDARGS----ILDSDGGLNGES----RGRPQEAVVA
AN5217.1  	DDDEE------------AASDGDIRGREE------EREPVPV
          	.::*               *.. : *. .        *.* .
```
